# Refracture of the cemented vertebrae after percutaneous vertebroplasty: risk factors and imaging findings

**DOI:** 10.1186/s12891-021-04355-w

**Published:** 2021-05-19

**Authors:** Yu-chao Xiong, Wei Guo, Fan Xu, Ci-ci Zhang, Zhi-ping Liang, Li Wu, Song Chen, Xu-wen Zeng

**Affiliations:** 1 Department of Radiology, Guangzhou Red Cross Hospital, Jinan University, 396 Tongfu Road, Guangzhou, 510220 Guangdong Province China; 2 Department of Radiology, Wuhan Third Hospital, Tongren Hospital of Wuhan University, 241 Liuyang Road, Wuhan, 430063 Hubei Province China

**Keywords:** Vertebroplasty, Risk factors, Magnetic resonance imaging, Spinal fractures, Bone cements

## Abstract

**Background:**

To determine the related imaging findings and risk factors to refracture of the cemented vertebrae after percutaneous vertebroplasty (PVP) treatment.

**Methods:**

Patients who were treated with PVP for single vertebral compression fractures (VCFs) and met this study’s inclusion criteria were retrospectively reviewed from January 2012 to January 2019. The follow-up period was at least 2 years. Forty-eight patients with refracture of the cemented vertebrae and 45 non-refractured patients were included. The following variates were reviewed: age, sex, fracture location, bone mineral density (BMD), intravertebral cleft (IVC), kyphotic angle (KA), wedge angle, endplate cortical disruption, cement volume, surgical approach, non-PMMA-endplate-contact (NPEC), cement leakage, other vertebral fractures, reduction rate (RR), and reduction angle (RA). Multiple logistic regression modeling was used to identify the independent risk factors of refracture.

**Results:**

Refracture was found in 48 (51.6%) patients. Four risk factors, including IVC (*P* = 0.005), endplate cortical disruption (*P* = 0.037), larger RR (*P* = 0.007), and NPEC (*P* = 0.006) were found to be significant independent risk factors for refracture.

**Conclusions:**

Patients with IVC or larger RR, NPEC, or endplate cortical disruption have a high risk of refracture in the cemented vertebrae after PVP.

## Background

Percutaneous vertebroplasty (PVP) is a minimally invasive technique for the treatment of vertebral compression fractures (VCFs). Most clinical studies [1–5] have reported that this treatment can provide immediate pain relief and biomechanical stability, and restore partial vertebral height. Despite these excellent clinical results, complications such as cement leakage, infection, embolism, fractures in the adjacent vertebrae, and refracture of previously treated vertebrae have been reported [6–10]. However, recompression in cemented vertebrae may lead to aggravation of the kyphotic deformity, vertebral height loss, and even compression of the spinal cord by vertebral body fracture, which usually requires further treatment [8, 9]. Some researchers [8, 11, 12] believe that cement distribution patterns may be an important predisposing factor to refracture. Kim [13] reported that the intravertebral cleft (IVC) might be a significant risk factor. Although research has highlighted many risk factors, refracture of the cemented vertebrae remains a controversial topic.

The treatment of refracture in cemented vertebrae remains challenging. The treatment strategy for vertebral fractures need to be changed when the risks of PVP outweighs the efficacy. Thus, the purpose of this study was to assess the related imaging findings and risk factors of patients who experienced refracture of the cemented vertebrae after PVP.

## Methods

### Patient selection

This retrospective cohort study was conducted from January 1, 2012 to January 1, 2019 in the spine surgery department of our hospital. The research program was approved by Institutional Review Board of Guangzhou Red Cross Hospital, and all procedures were performed according to the Declaration of Helsinki. All patients received written informed consent before operation.

A total of 1303 patients who were diagnosed with VCF (T4-L5) receiving single level PVP were enrolled in this study. Patients who met the following criteria were excluded:

(i) pathological vertebral fractures secondary to tumor, severe inflammation, or long-term corticosteroid use;
(ii) patients without available radiographs or magnetic resonance imaging (MRI);
(iii) No history of PVP surgery;
(iv) patients with neurologic deficits;
(v) follow-up time less than 24 months;
(vi) patients with hyperparathyroidism, hyperthyroidism, or other bone metabolic diseases

The inclusion criteria were as follows:

(i) patients who had plain films preoperatively, immediately after surgery, and at the final follow-up;
(ii) patients who underwent MRI preoperatively and at last follow-up;
(iii) follow-up period of at least 2 years;
(iv) patients with a bone density scan before the PVP;
(v) single-level symptomatic VCF treated with PVP;

Based on these criteria, a total of 93 patients were enrolled in our study (75 women, 18 men).

### Operative procedure

All patients received bilateral or unilateral PVP in the prone position under the guidance of C-arm fluoroscopy after local anesthesia (1% lidocaine). According to Jensen’s technique [14], under C-arm fluoroscopic control, 11-gauge or 13-gauge bone biopsy needles were entered the pedicle in a slightly descending manner or parallel to the superior and inferior edges of the pedicle. The needle was positioned in the optimal position as confirmed by C-arm, that is, the tip reached the anterior third of the vertebral body and the middle height of the midline. After the stylet was removed from the trocar, a formulated polymethylmethacrylate (PMMA) mixture was instilled, filling the fractured bone. The cement injection process was performed slowly, and strictly monitored under C-arm fluoroscopy in the lateral plane to avoid cement leakage. The bone cement filled the fractured vertebrae in the anterior third of the vertebral body as much as possible to form an effective mechanical column. The injection was immediately stopped when cement leakage was seen in the segmental vein, adjacent intervertebral disk, epidural space, or epidural vein. After PVP, all patients were allowed to ambulate the day after surgery.

### Imaging examinations

Prior to PVP and at least 24 months of follow-up, all patients underwent spinal MR examinations of the spine supine position. The MR examinations were performed with a 1.5-T (Siemens Avanto) imager with the following sequences: a sagittal T1- weighted spin-echo sequence (TR, 535 ms; TE, 11 ms), a sagittal T2-weighted spin-echo sequence (TR, 3500 ms; TE, 90 ms), and a spectral attenuated inversion recovery (SPAIR) sequence (TR, 3500 ms; TE, 90 ms). Prior to PVP and within 2 weeks of PVP and following underwent anteroposterior and lateral radiographs of the spine supine position.

### Radiological assessment

All images were analyzed during a time span of 2 weeks. Images were randomly evaluated by two experienced musculoskeletal radiologists in a random order, each blinded to the clinical information. In our study, the inter observer correlation coefficient (ICC) of all radiology parameters was excellent (ICC > 0.85). A consensus was reached when two observers disagreed on the first reading. Face-to-face training was conducted before the study.

#### Anatomical locations of the involved vertebrae

The anatomical locations of the involved vertebrae were divided into two groups: vertebrae outside the thoracolumbar junction (from T4 to T9 or L3 to L5) and vertebrae at the thoracolumbar junction (from T10 to L2) [15].

#### Intravertebral cleft (IVC)

The IVC was detected as an area of signal loss (gas-containing space) or showing marked hyperintensity (fluid collection) on the preoperative sagittal T2-weighted images [15, 16].

#### Endplate cortical disruption

Endplate cortical disruption was determined as evident discontinuation in the cortical endplate as seen on the preoperative sagittal T2/T1-weighted images [17].

#### Kyphotic angle (KA) (Cobb’s angle) and reduction angle (RA)

KA was defined as the angle between the upper endplate of the upper vertebra and the lower endplate of the lower vertebra. At L5, the KA was defined as the angle between the upper endplate of L4 and the upper endplate of the sacral vertebra. RA was calculated as the difference between preoperative and immediate postoperative KA [18].

#### Wedge angle (WA)

WA was defined as the angle between the upper endplate line and the lower endplate of the fractured vertebra.

#### Vertebral compression rate (CR), reduction rate (RR) and compression rate increase (CRI) (Fig. 1)

CR refers to the ratio of vertebral height of the fractured vertebrae to the average vertebral height of the upper and lower vertebrae at the same site [18]. At L5, CR was the ratio of L4 vertebral height to L5 vertebral height at the same site. RR was calculated as the difference between preoperative and immediate postoperative CR [18].CRI was defined as the difference in CR between immediately after surgery and the last follow-up.

**Fig. 1:**
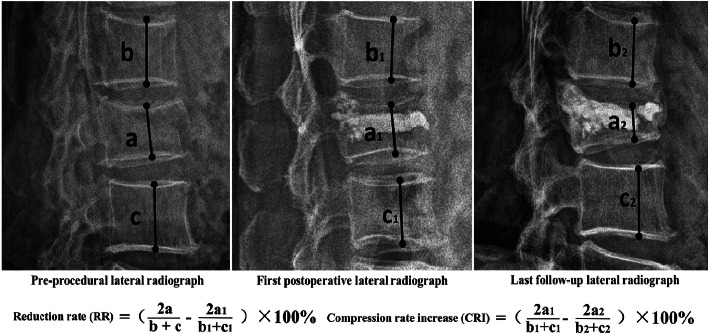
The reduction rate (RR) and compression rate increase (CRI) were calculated as above

#### Cement leakage

Cement leakage was defined as any cement present in the space beyond the cortical margin [19].

#### Non-PMMA-endplate-contact (NPEC)

NPEC was defined as postoperative plain radiographs showing that the injected PMMA did not come into contact with the upper and lower endplates [20]. The patterns of NPEC were classified as NPEC on the upper endplate, NPEC on the lower endplate, NPEC on the upper and lower endplates, and no NPEC on anteroposterior and lateral radiography of the treated vertebra [21].

### Clinical data analysis

The medical records were retrospectively analyzed to collect the use of anti-osteoporosis drug. Medicament for the treatment of osteoporosis include zoledronic acid, calcium and vitamin D supplements. Effective anti-osteoporosis therapy needs to meet the minimum drug ownership rate of 80% within 6 months [22]. Patient demographics, including gender, age, interval (the period between the start of new back pain related to MRI-confirmed fracture and the time of PVP), other vertebral fractures, surgical approach, bone mineral density (BMD), and cement volume were also analyzed.

### Statistical analysis

All statistical analyses were performed using statistics software (SPSS, Chicago, IL, USA). *P* < 0.05 indicated a statistically significant difference. Logistic regression univariate and multivariate analyses were used to assess the risk factors for refracture of the cemented vertebrae after PVP. The possible risk factors with *P* value less than or equal to 0.10 in univariate analysis were input into the final multivariate logistic regression model. After adjusting other risk factors, the significance of each risk factor on refracture was tested.

## Results

In total, 93 patients (refracture group, *n* = 48; non-refracture group, *n* = 45) were reviewed. Patients in the refracture group were followed for 1.2–25.9 months (mean, 9.3 months; median, 11.2 months). In the refracture group, the CRI was 15.1–40.2% (mean, 23.87%; standard deviation (SD), 7.89%). Patients in the non-refracture group were followed for 24.8–46.6 months (mean, 33.2 months; median, 38.4 months).

Univariate analysis revealed that IVC (*P* < 0.001), endplate cortical disruption (*P* = 0.026), reduction rate (*P* < 0.001), NPEC (*P* < 0.001), and kyphotic angle (*P* = 0.014) were significant factors for refracture of the cemented vertebrae after PVP (Table 1). On multivariate analysis, however, IVC (*P* = 0.005; odds ratio, 27.12; 95% confidence interval [CI]: 2.67, 275.38), endplate cortical disruption (*P* = 0.037; odds ratio, 3.23; 95% confidence interval [CI]: 1.07, 9.75), larger RR (*P* = 0.007; odds ratio, 2.94; 95% confidence interval [CI]: 1.33, 6.47), and NPEC (*P* = 0.006; odds ratio, 1.99; 95% confidence interval [CI]: 1.23, 3.24) showed significance after adjustment for other variables (Table 2).

**Table 1:** Univariate analysis: clinical factors and imaging finds in the refracture and non-refracture groups

clinical factors and imaging finds	Refracture(n = 48)	Non-refracture(n = 45)	*P* value
Age (years)	79.65 ± 7.90	78.29 ± 7.21	0.303
Grender			
Men	8	10	
Women	40	35	0.498
Fracture location			
Thoracolumbar	33	36	
Non-thoracolumnar	15	9	0.215
BMD(g/cm^2^)	0.664 ± 0.15	0.73 ± 0.16	0.686
kyphotic angle (^o^)	17.56 ± 9.51	12.58 ± 9.64	0.014
Wage angle (^o^)	10.88 ± 6.28	10.20 ± 5.01	0.204
IVC			
Present	18	1	
Absent	30	44	<0.001
Endplate cortical disruption			
Present	27	15	
Absent	21	30	0.026
Bone cement volume (ml)	3.13 ± 0.709	3.01 ± 0.663	0.440
Surgical approach			
Left	5	4	
Right	38	39	
Bilateral	5	2	0.605
NEPC			
Present on lower endplate	14	7	
Present on upper endplate	7	10	
Present on upper and lower endplate	26	16	
Absent	1	12	<0.001
Leakage of bone cement			
Present	13	9	
Absent	35	36	0.422
other vertebral fractures			
Adjacent vertebral fracture	8	5	
Non-adjacent vertebral fracture	21	11	
No	19	29	0.134
Reduction rate (%)	11.92 ± 11.18	4.44 ± 5.18	<0.001
Reduction angle (°)	4.02 ± 3.89	4.76 ± 6.28	0.491
Effect anti-osteoporotic therapy			
Yes	34	33	
No	14	12	0.486

**Table 2:** Outcome of multivariate logistic regression analysis

	OR (95% CI)	*P* value
Endplate cortical disruption	3.23 (1.07–9.75)	0.037
IVC	27.12 (2.67–275.38)	0.005
RR (%)	2.94 (1.33–6.47)	0.007
NPEC	1.99 (1.23–3.24)	0.006

Data were analyzed with logistic regression. Multivariable analysis adjusted for Endplate cortical disruption, IVC, RR, and NPEC

*CI* Confidence interval, *OR* Odds ratio

Analysis of the relationship between endplate cortical disruption and the displacement of the anterior edge of the vertebral body found that the anterior movement of the vertebral body was 3.12 ± 2.62 mm for with endplate cortical disruption, and 1.67 ± 2.18 mm for without endplate cortical disruption (*P* < 0.05).

## Discussion

Researchers have not uniformly described the loss of vertebral height and the criteria for unified diagnosis of height loss in cemented vertebrae after PVP. He [12] and Kim [20] described ‘recompression’ of previously treated vertebrae. The term ‘recompression’ might be confused with additional loss of vertebral height, including osteoporosis [23]. Heo [9] and Yu [18] reported using recollapse to describe the loss of the same vertebrae after PVP. The term ‘recollapse’ might be misunderstood cement block cracked [12]. In the present study, there was vertebral bone marrow edema, and the loss of vertebral height only in the bony vertebra, not in the cement mass. For these reasons, we recommend using the term ‘refracture’ to describe this condition. In previous studies, recompression or refracture of augmented vertebrae were defined as a height reduction of 1 mm or 4 mm on follow-up radiographs [12, 15, 20, 24–27]. Due to the magnification ratio on radiographs, the measurement of height loss can easily lead to incorrect evaluation. In addition, several long-term studies of patients who were post-vertebral augmentation have showed that in up to 30% of patients have a gradual decrease in vertebral body height of 10 to 15% after PVP between 12 and 24 months [28]. Thus, in this study, the criterion of 15% decrease in height [18] and presence of vertebral bone marrow edema was adopted.

Although the risk factors for refracture in cemented vertebrae after PVP have been previously reported [8, 11], to our knowledge this is the first study to report the risk factors and imaging findings of refracture based on bone marrow edema as a diagnostic basis.

The incidence of refracture in cemented vertebrae was 3.68%(48/1303) in this study, which was approximately consistent with the findings in previous studies, which ranged from 0.56 to 27.63% [8, 9, 18, 24, 29]. However, the author infers that the true incidence of refractures should be higher than the expected data in the present study. This is because some patients with refracture did not seeking medical services and the present study used rigorous inclusion and exclusion criteria and grouping criteria.

We evaluated the risk factors for refracture in cemented vertebrae. IVC showed a statistically significant relationship to refracture (Table 2), which corresponds with many previous studies [8, 9, 11, 18, 30, 31]. IVC is a risk factor for refracture in cemented vertebrae and can be explained by two factors; namely, the IVC factor and secondary changes caused by IVC.

**Fig. 2:**
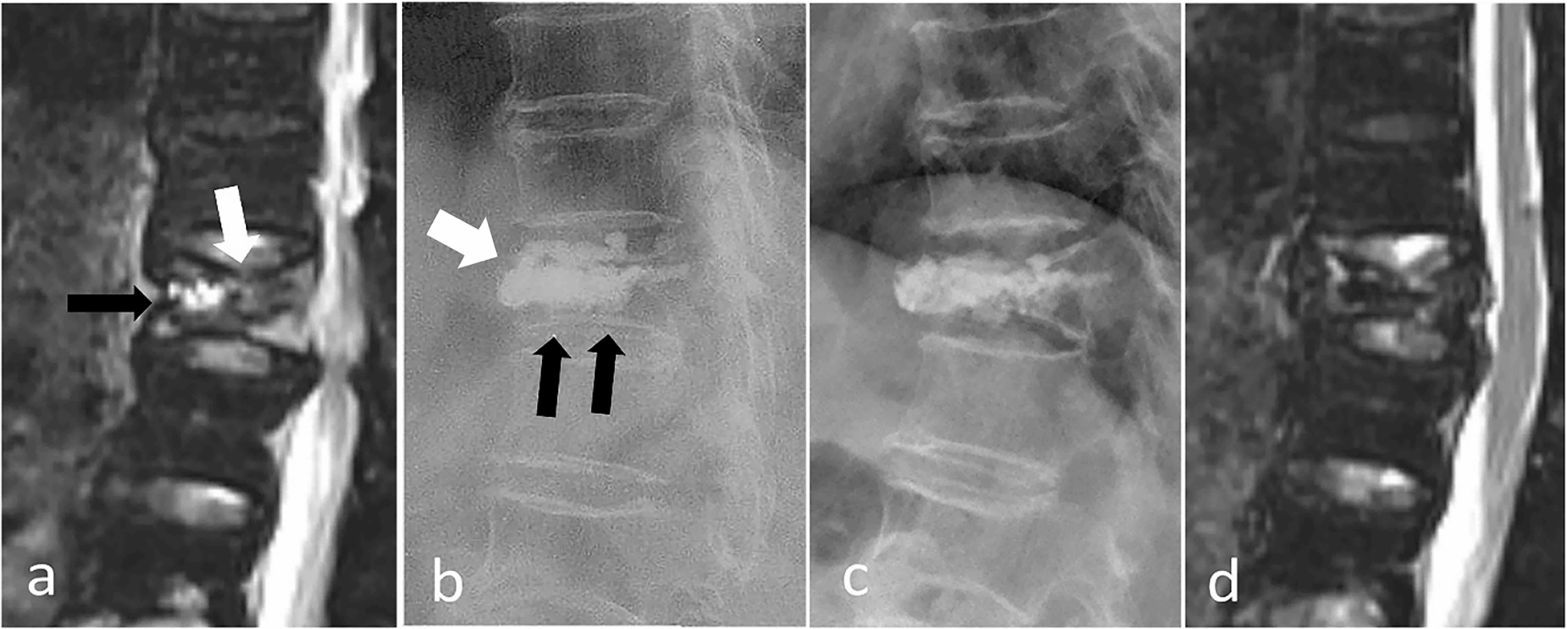
An 82-year-old woman with refracture after PVP. MRI and lateral plain X-ray of an 82-year-old woman with a T11 compression fracture. **a** Sagittal SPAIR showed a T11 compression fracture with bone marrow edema, IVC (black arrow), and endplate cortical disruption (white arrow). **b** Postoperative X-ray showed the cemented vertebrae with NPEC on the lower endplate (black arrow) and displacement of the anterior edge of the vertebral body (white arrow). **c**-**d** MRI and lateral X-ray at 5 months after PVP showed loss of height of T11 and bone marrow edema

**Fig. 3:**
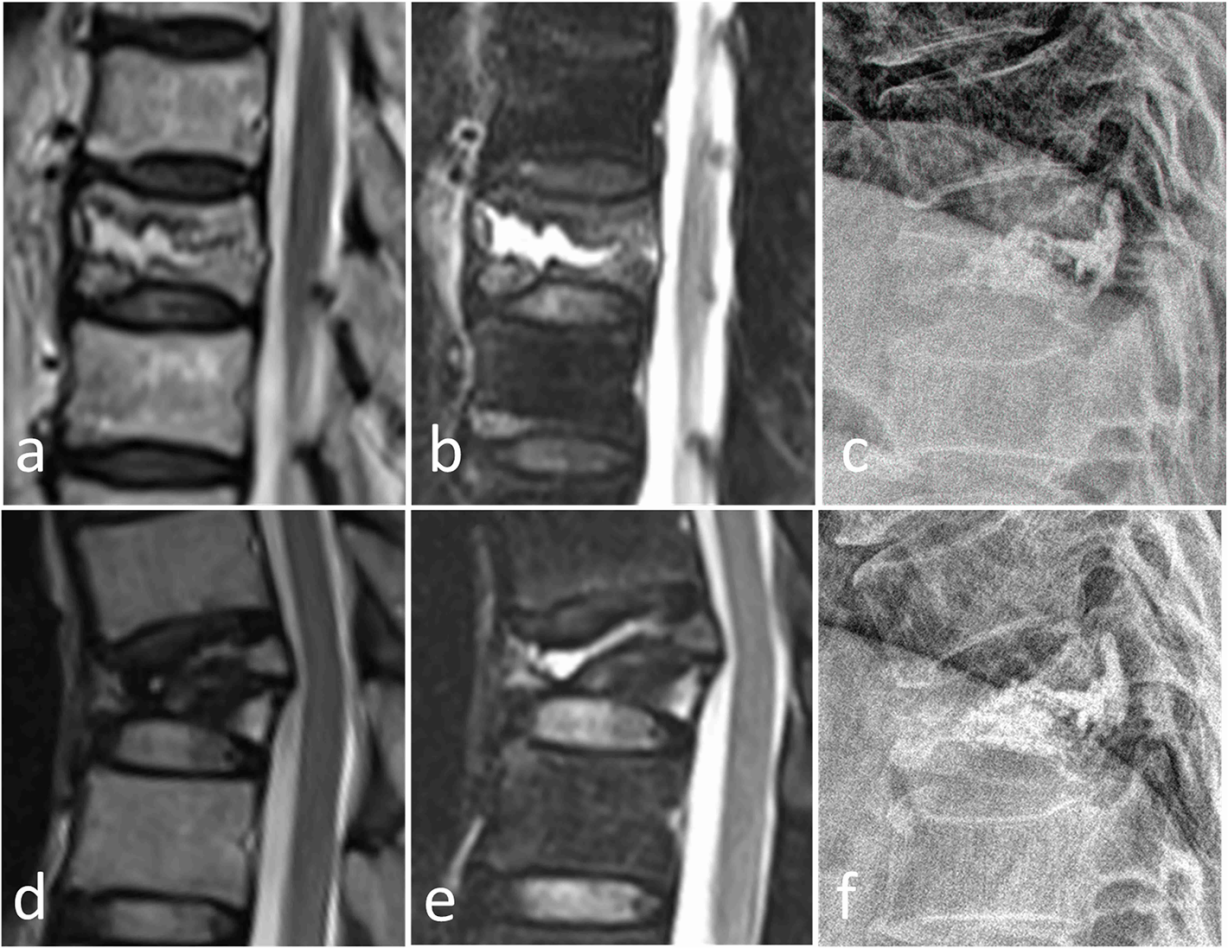
A 59-year-old man with refracture after PVP. Preoperative T2W and SPAIR images (**a**, **b**) showed a T10 compression fracture with IVC. Postoperative lateral radiographs (**c**) after 2 days showed NPEC on the lower and upper endplates. The T2WI, SPAIR image, and lateral radiograph 5 months after PVP (**d**-**f**) showed refracture of the cemented T10 vertebra with loss of height and bone marrow edema

With regard to the IVC factor, IVC provides radiological evidence of osteonecrosis [29, 32, 33]. Osteonecrosis of the involved vertebrae would progress after PVP, which would eventually weaken the structural rigidity of the vertebral body and result in refracture of the remainder of the vertebral body (Figs. 2 and 3) [9]. In addition, the fractured vertebrae with IVC was usually a solid lump cased volumetric pressure effect that may aggravate the process of osteonecrosis [9]. Heo et al. [9] also reported that the timing of PVP is of great importance for patients with IVC, given that it is likely unfavorable during the early phase of osteonecrosis. Premature PVP may cause collateral vessels in necrotic bone to fail to form.

With regard to secondary changes caused by the IVC factor, IVC was related to cement distribution pattern [9, 12, 18] and vertebral height restoration [34].

When bone cement is injected, it often enters the low-pressure zone (IVC zone), which causes the bone cement to form solid lump cement, making it easier to form NPEC (Fig. 2). In our study, the incidence of NPEC was significantly higher in patients with IVC (94.74%) than without IVC (78.38%). Further analysis also indicated that NPEC was a risk factor associated with refracture (Table 2). Zhang [24] found that patients without NPEC had a lower risk of recompression compared with patients with NPEC on the upper and lower endplates. Hou [25] found that the smaller the distance between PMMA and the endplate, the lower the incidence of recompression. Heo [20] found that recompression occurred in unsupported areas of PMMA. Our findings are consistent with previous research. Bone cement is in contact with both the upper and lower endplates, so it can provide a better support in the vertical direction since the load is transmitted through both the upper and lower endplates, which are harder in nature. When bone cement had NPEC, the load did not transmitted through the cementless area, resulting in a stress shielding effect, so the bone cement may serve to concentrate stress on the surrounding fragile bones and lead to refracture.

In our study, compared with the VCFs without IVC, the height of the vertebral body with IVC increased from 7.19 to 12.63%. Michael [34] also reported that vertebroplasty increased the height of the fractured vertebrae, and these effects were most remarkable in fractured vertebrae with IVC. Further analysis indicated that RR was a risk factor associated with refracture (Table 2). Lin [11] also found that cemented vertebrae with significant vertebral height restoration after PVP were prone to refracture. Too much recovery of the vertebral body may lead to increased tension of the paravertebral soft tissue, which may lead to increased mechanical load on the enlarged vertebrae or more unstable fractures. Consequently, the risk of refracture of involved vertebrae increased with a greater degree of height restoration.

Overall, IVC affected RR and NPEC, but RR and IVC were not all dependent on the impact of IVC. The NPEC, IVC, and RR were independent risk factors for refracture in cemented vertebrae after PVP.

**Fig. 4:**
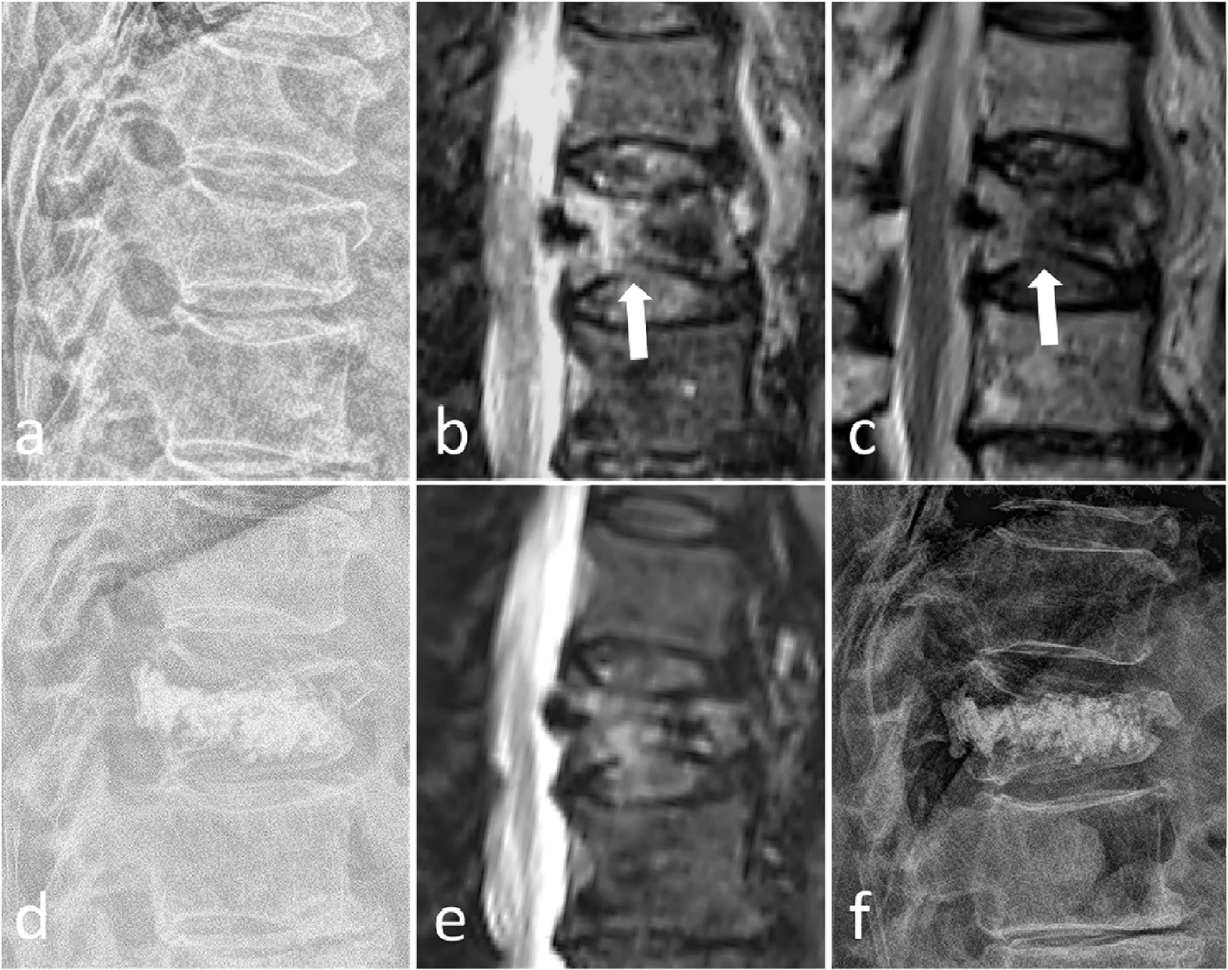
A, 85-year-old woman with refracture after PVP. Preoperative lateral radiographs, SPAIR, and T2W images (**a**-**c**) showed a T12 compression fracture with endplate cortical disruption (black arrow). Postoperative lateral radiographs (**d**) after 1 day showed NPEC on the lower and upper endplates. The SPAIR image and lateral radiograph 6 months after PVP (**e**, **f**) showed refracture of the cemented T12 vertebra with loss of height and bone marrow edema

**Fig. 5:**
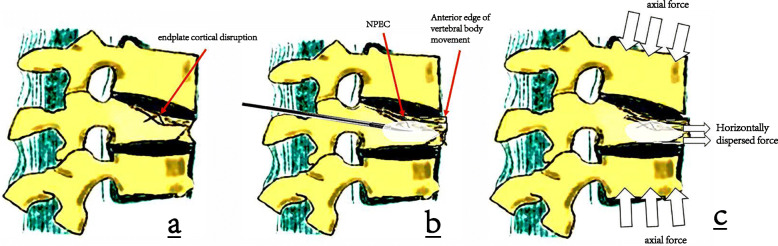
Schematic diagram of refracture caused by endplate cortical disruption. Shows the presence of endplate cortical disruption (**a**). When the bone cement is injected, the displacement of the anterior edge of the vertebral body offsets the force of the bone cement to diffuse into the trabecular bone and causes NPEC (**b**). When subjected to axial pressure, part of the force will migrate to the horizontal direction, and the vertebral body is weak against the horizontal direction, resulting in refracture (**c**)

In our study, endplate cortical disruption was also an independent risk factor for refracture in cemented vertebrae after PVP (Table 2) (Fig. 4). We explained the occurrence of refracture after PVP as a biomechanical model (Fig. 5). When there is endplate cortical disruption, the anterior edge of the vertebral body will move forward in the process of bone cement injection (Fig. 2). Anterior vertebral displacement causes two consequences. On one hand, the axial force of the vertebral body will be partially dispersed laterally, and the vertebral body is weak against lateral pressure. On the other hand, the fractured vertebral body cannot be sufficiently filled with bone cement, because the displacement of the anterior edge of the vertebral body can offset the force of the bone cement to diffuse into the trabecular bone. As a result, the cemented vertebrae with endplate cortical disruption is more vulnerable to refracture.

Previous research found that BMD was a risk factor for refracture after surgery [31, 35–37]. However, in our study BMD was not a risk factor for refracture. We speculate that this is due to the fact that this study used bone marrow edema as a diagnostic criterion for refracture. Villarraga [37] found that the loss of height in cemented vertebrae was the natural development of osteoporosis concluded from finite element model analysis, so the loss of height in cemented vertebrae caused by osteoporosis may not cause bone marrow edema.

Although some studies [1–5] reported that PVP could provide significantly pain relief in patients with VCFs, more and more studies [38, 39] does not support significant clinically benefits from PVP comparaed with placebo. Refarcture in cemented vertebrae may be one of the reasons why pain relief is not better than placebo. In our opinion, looking for strategies for poor clinical effects of PVP provides clinicians with a pragmatic method of how to best treat patients.

This study had several limitations. First, it was a retrospective study with a single center and relatively small sample size, and a prospective, multi-center studies with a larger sample size are required to ensure the universality of our conclusions. Second, two-dimensional X-ray was used to determine the NPEC. In these images, NPEC may be underestimated. To accurately assess NPEC, three-dimensional CT scans will help. Finally, although there is no significant difference in effective anti-osteoporosis therapy between the refracture group and the non-refracture group, a large proportion of patients receiving effective anti-osteoporosis treatment in this study may lead to bias.

## Conclusions

Four independent risk factors were significantly associated with refracture of the cemented vertebrae after PVP, including intravertebral cleft, non-PMMA-endplate-contact, increased reduction rate, and endplate cortical disruption. Therefore, the current surgical methods and treatment strategies may need to be adjusted on the basis of the risk factors of patients.

## Data Availability

The datasets used and analysed during the current study are available from the corresponding author on reasonable request.

## Abbreviations

PVP: Percutaneous vertebroplasty
VCFs: Vertebral compression fractures
BMD: Bone mineral density
IVC: Intravertebral cleft
KA: Kyphotic angle
NPEC: Non-PMMA-endplate-contact
RR: Reduction rate
RA: Reduction angle
PMMA: Polymethylmethacrylate
MRI: Magnetic resonance imaging
WA: Wedge angle
CR: Compression rate
CRI: Compression rate increase
CI: Confidence interval
